# Evoked Resonant Neural Activity in subthalamic local field potentials reflects basal ganglia network dynamics

**DOI:** 10.1016/j.nbd.2023.106019

**Published:** 2023-03

**Authors:** Christoph Wiest, Shenghong He, Benoit Duchet, Alek Pogosyan, Moaad Benjaber, Timothy Denison, Harutomo Hasegawa, Keyoumars Ashkan, Fahd Baig, Ilaria Bertaina, Francesca Morgante, Erlick A. Pereira, Flavie Torrecillos, Huiling Tan

**Affiliations:** 1 Medical Research Council Brain Network Dynamics Unit, Nuffield Department of Clinical Neurosciences, University of Oxford, Oxford, UK; 2 Department of Neurosurgery, King’s College Hospital, Denmark Hill, London, UK; 3 Neurosciences Research Centre, Molecular and Clinical Sciences Institute, St. George’s, University of London, London, UK; 4 Neurology Department, Neurocenter of Southern Switzerland, Ente Ospedaliero Cantonale, Lugano, Switzerland

**Keywords:** Parkinson’s disease, Deep brain stimulation, Evoked resonant neural activity, Adaptive DBS, Local field potentials, Subthalamic nucleus

## Abstract

Evoked resonant neural activity (ERNA) is induced by subthalamic deep brain stimulation (DBS) and was recently suggested as a marker of lead placement and contact selection in Parkinson’s disease. Yet, its underlying mechanisms and how it is modulated by stimulation parameters are unclear. Here, we recorded local field potentials from 27 Parkinson’s disease patients, while leads were externalised to scrutinise the ERNA. First, we show that ERNA in the time series waveform and spectrogram likely represent the same activity, which was contested before. Second, our results show that the ERNA has fast and slow dynamics during stimulation, consistent with the synaptic failure hypothesis. Third, we show that ERNA parameters are modulated by different DBS frequencies, intensities, medication states and stimulation modes (continuous DBS vs. adaptive DBS). These results suggest the ERNA might prove useful as a predictor of the best DBS frequency and lowest effective intensity in addition to contact selection. Changes with levodopa and DBS mode suggest that the ERNA may indicate the state of the cortico-basal ganglia circuit making it a putative biomarker to track clinical state in adaptive DBS.

## 1 Introduction

Deep brain stimulation (DBS) to the subthalamic nuclei (STN) is an effective treatment option for advanced Parkinson’s disease (PD) and yet its underlying mechanisms are still a matter in dispute (Chiken and Nambu, 2016). To help unravel the intricacies of DBS, we take the approach of studying DBS-induced changes in the STN. Beta power suppression is the clearest and most robust spectral change during STN-DBS, which was linked with therapeutic effects of stimulation (Kühn et al., 2008; Whitmer et al., 2012). Another prominent feature of STN-DBS is evoked resonant neural activity (ERNA). The ERNA was initially reported in the time domain as a high-amplitude, high-frequency oscillation with underdamped characteristics (Sinclair et al., 2018). In the frequency domain, the ERNA presents as a high-frequency oscillation, which decreases in frequency over the first minute of stimulation to the STN (Wiest et al., 2020). However, Ozturk et al. challenged the view that ERNA in time and frequency domain actually represent the same activity by showing that ERNA can be observed in the time domain at non-therapeutic DBS frequencies, but not in the frequency domain (Ozturk et al., 2021). In a first step, we seek to compare the two ERNA analysis approaches to show that ERNA quantified in time and frequency domain reflect the same activity.

The ERNA has recently been suggested as a marker for intraoperative lead placement even during general anaesthesia and postoperative contact selection (Sinclair et al., 2018; Xu et al., 2022), but it is yet unclear what the underlying mechanism is and to what extent it is modulated by dopaminergic medication or other DBS settings. It was previously shown that pallidal and STN neurons fire rhythmically and time-locked to DBS pulses (Bar-Gad et al., 2004; Hashimoto et al., 2003), which led to the information jam theory of DBS (a pathological rhythm is drowned by a physiological rhythm) and may have been a first hint at the mechanism underlying the ERNA. More recently, a computational model suggested that the ERNA may be initiated through direct activation of cortico-subthalamic fibres and subsequently sustained by reciprocal connections between the external globus pallidus (GPe) and STN (Schmidt et al., 2020). Therefore, the ERNA may indicate the connectivity and circuit dynamics of the STN-GPe network. In a second step, we seek to test if ERNA parameters and by inference STN-GPe circuit dynamics change with dopaminergic medication and we suspect to observe corresponding changes with therapeutic DBS frequencies and intensities.

## 2 Methods

### 2.1 Consent, regulatory approval, patient selection and clinical details

This protocol was approved by the Health Research Authority UK and the National Research Ethics Service local Research Ethics Committee (IRAS: 46576). Patients with PD were recruited for LFP recording at St. George’s University Hospital NHS Foundation Trust, London, King’s College Hospital NHS Foundation Trust, London, and University Medical Center Mainz. Written informed consent was obtained in line with the Declaration of the Principles of Helsinki. 27 patients with idiopathic PD undergoing bilateral STN-DBS surgery were included in the study. Patients were selected by an interdisciplinary team of movement disorder neurologists, neuropsychologists, functional neurosurgeons and DBS nurses if they met the UK Parkinson’s Disease Society Brain Bank Diagnostic Criteria for diagnosis of PD (Hughes et al., 1992). The average age at the time of recording was 60.42 ± 1.22 years (mean ± SEM) with average disease duration of 10.85 ± 0.98 years. 9 patients were recorded bilaterally, resulting in a total of 36 STNs included in the study. During the recordings, an experienced neurologist was present to screen for unwanted side effects of stimulation. Clinical details are summarised in Table 1. Baseline motor function in the ON and OFF medication state were assessed pre-operatively in 25 patients and on the day of the recording in 2 patients using part III of the Unified Parkinson’s Disease Rating Scale motor subscale (UPDRS-III).

### 2.2 Surgery and lead localisation assessment

**Figure 1:**
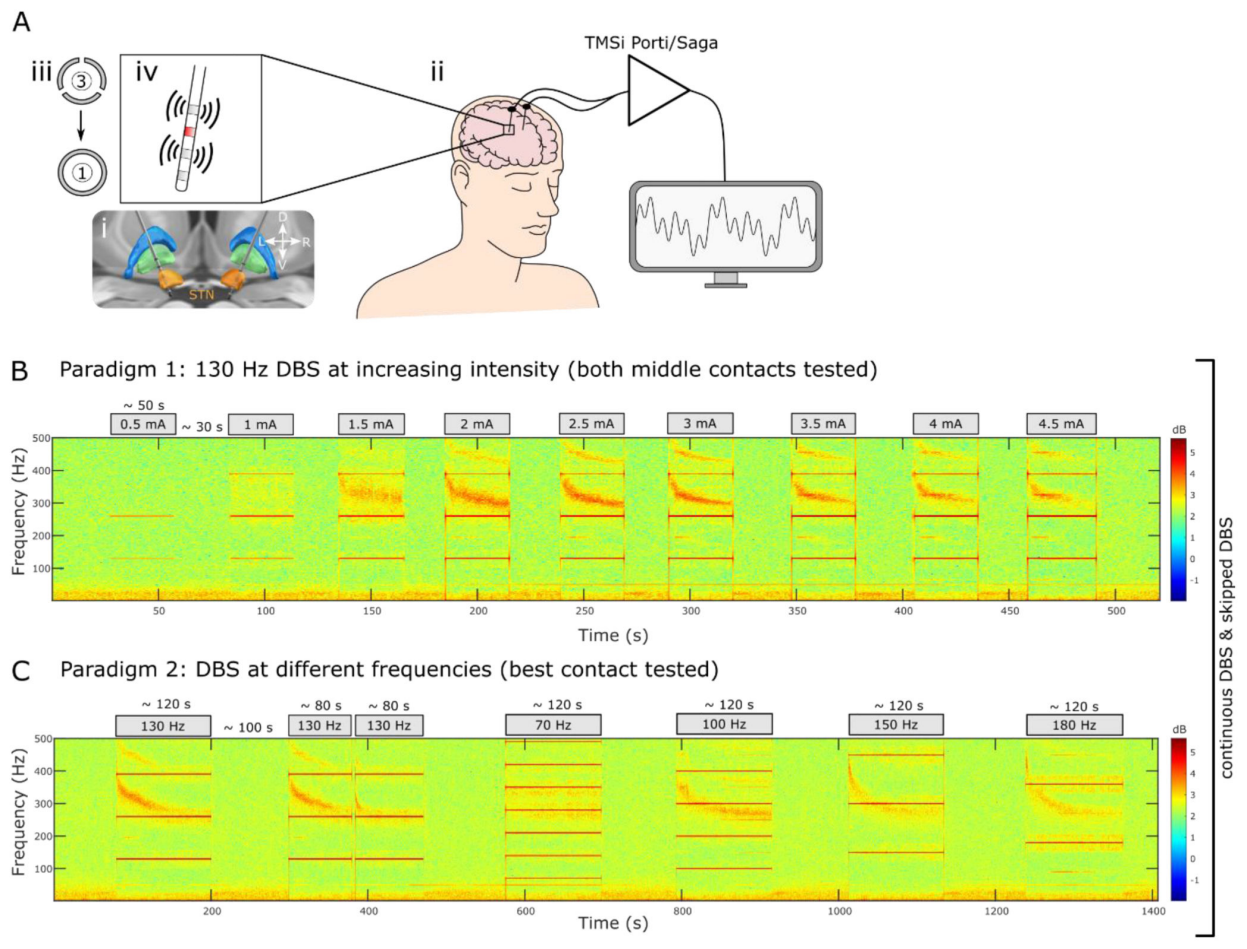
Recording setup and experimental paradigm. **A.** Bilateral electrodes were implanted in the subthalamic nucleus (STN) (i) and externalised (ii). Directional contacts were fused to form a ring contact (iii) and stimulation was applied to either of the two middle contact levels (red), which allowed recording from the adjacent contact levels in a bipolar montage (iv). **B.** Spectrogram of the bipolar channel that surrounds the stimulation contact (Paradigm1, shown for one example hemisphere: Patient 6 left). DBS intensity was increased in steps of 0.5 mA. **C.** The contact level and current that elicited the largest ERNA amplitudes without side effects in paradigm 1 were chosen to test the effect of different stimulation frequencies on the ERNA in paradigm 2. Both paradigms (B+C) were tested with continuous DBS and skipped DBS (1 pulse was skipped every second during DBS and after DBS blocks the ERNA was probed with bursts of 10 pulses spaced 1 second apart, see Methods).

The surgical target was STN. DBS systems from 3 manufacturers were used (see Table 1): Medtronic Inc. Neurological Division, USA (either a quadripolar non-directional lead (model 3389) or octopolar directional lead with 1-3-3-1 configuration (SenSight™)), Boston Scientific, USA (either an octopolar directional lead with 1-3-3-1 configuration (Vercise™ model DB-2202) or new experimental leads with 16 contacts and either 3-3-3-3-1-1-1-1 or 3-3-3-3-3-1 configuration (Cartesia™ X/HX)) and Abbott Inc., USA (octopolar directional leads with 1-3-3-1 configuration (Infinity™ model 6172ANS)). Directional contacts were fused to form a ring contact (Figure 1Aiii). Electrodes were implanted under general anaesthesia using frame-based stereotaxy, MRI guidance and CT-fusion verification (St. George’s) or during awake surgery with additional intra-operative stimulation testing (King’s). The implanted leads were connected to temporary lead extensions and externalised through the left temporal or frontal scalp. Assessment of contact localisation was blinded to the electrophysiological data, by co-registration of immediate post-operative CT with pre-operative MRI using the open-source LEAD-DBS toolbox (Horn et al., 2019). Lead placement for an example patient is shown in Figure 1Ai.

### 2.3 Stimulation and data recording

Recordings were made between 2 and 6 days postoperatively (Table 1), while electrode leads were still externalised and before implantation of the subcutaneous pulse generator. Patients performed the experiments either ON dopaminergic medication and/or after overnight withdrawal as indicated in Table 1. Monopolar high-frequency stimulation was only tested at the 2 middle contacts (the 2 lowest bipolar montages in STN if Cartesia X/HX was implanted) to allow bipolar LFP recordings from the two adjacent contacts (Figure 1Aiv). A self-adhesive electrode (Pals, Nidd Valley Medical Cordon, UK) attached to the patients’ back served as a reference for stimulation, which was delivered using a highly-configurable custom-built neurostimulator. Stimuli comprised symmetric, constant-current, biphasic pulses (60 μs per phase, negative phase first). Stimulation was applied at different intensity and frequency, according to a protocol described below. LFPs were amplified and sampled at 4096 Hz using a CE-marked multi-channel amplifier (TMSi Saga, TMSi International, Netherlands) or at 2048 Hz using a TMSi Porti (TMSi International, Netherlands) and custom-written software developed using the C programming language (Figure 1Aii). The ground electrode was placed on the forearm.

### 2.4 Experimental paradigm

**Figure 2:**
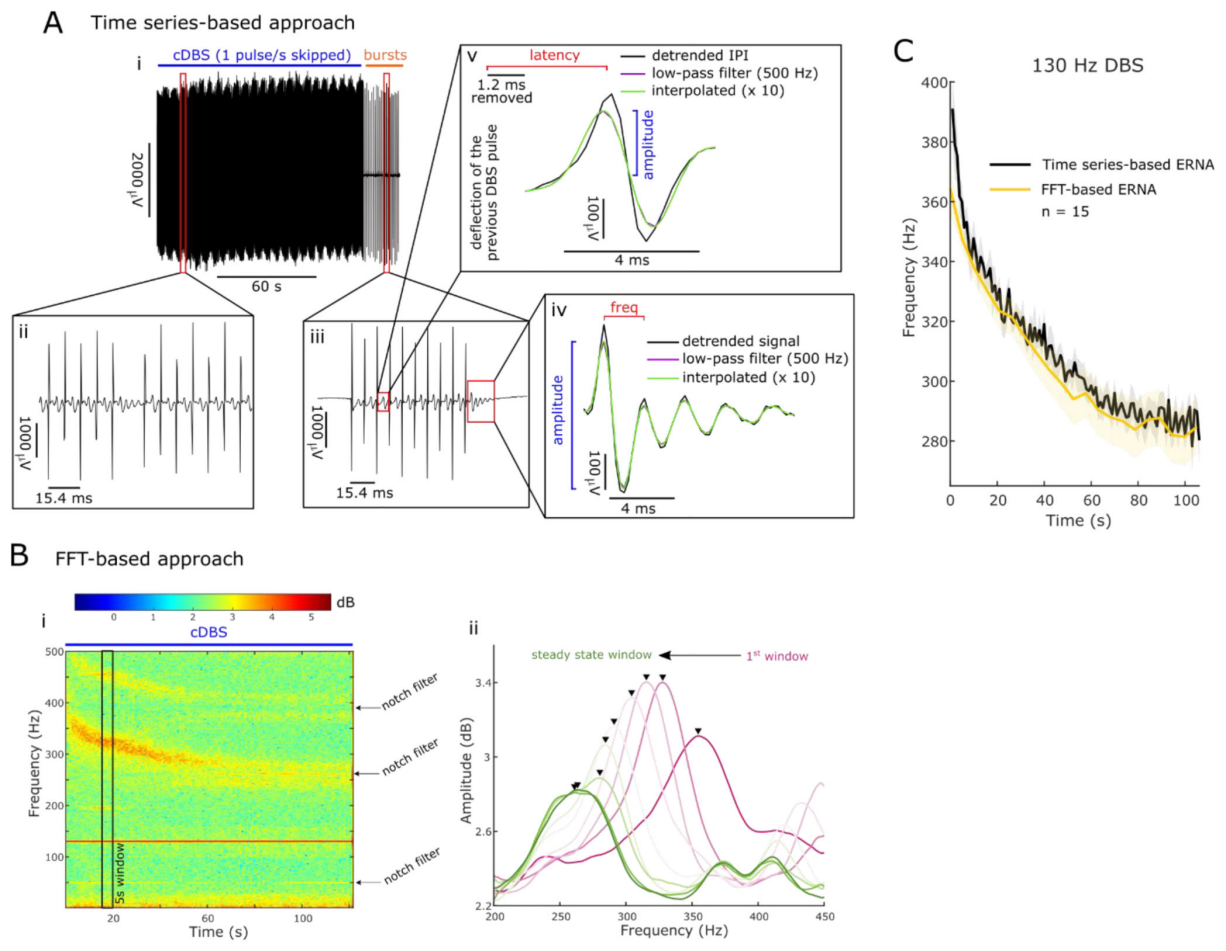
Time series and FFT-based ERNA represent the same activity. **A.** Skipping 1 DBS pulse per second (i) created an inter-pulse interval (IPI) that is twice as long (ii; 15.4 ms when DBS at 130 Hz). After DBS blocks, the ERNA was probed with 1 burst per second (each burst consisted of 10 pulses) for ~ 20 s (iii). This allowed us to extract ERNA frequencies and amplitudes based on the time series (iv) during and after a DBS block. In addition, we also extracted ERNA amplitudes and latencies after every pulse (v). **B.** Based on the spectrogram (short-time FFT; i), we calculated the average PSD for consecutive non-overlapping 5 s windows and tracked the ERNA as the largest peak between 200 and 450 Hz over time (ii). **C.** ERNA frequency dynamics calculated based on the time series and FFT-based approach (mean ± SEM) were not significantly different (permutation test for consecutive time points), which suggests they represent the same underlying phenomenon.

During the experiments, patients were comfortably seated in an armchair. We tested 3 different paradigms. Paradigm 1 was designed to test the effect of increasing stimulation intensity on the ERNA. 11 participants (15 hemispheres, Patients 1-11 see Table 1) were recorded in paradigm 1, where each of the 2 middle contacts was stimulated with increasing intensity from 0.5 mA in steps of 0.5 mA until either 4.5 mA or side effect threshold was reached (Figure 1B). Data from 2 hemispheres was excluded since no ERNA was detectable. DBS was applied continuously at 130 Hz in consecutive blocks that lasted on average 34.16 ± 1.37 s for each intensity level and were separated by resting periods of 31.96 ± 2.06 s. After paradigm 1, LFP spectrograms were visually inspected and waveforms were quantified to select the contact and current that elicited the largest ERNA amplitudes for paradigm 2 (Figure 1B, DBS intensity listed in Table 1). The same participants were recorded in paradigm 2, which helped to test the effect of stimulation frequency on the ERNA. In paradigm 2, we applied seven continuous DBS blocks at different frequencies (70, 100, 130, 150 and 180 Hz) according to the order and times indicated in Figure 1C. Both paradigms 1 and 2 were repeated using “skipped pulse DBS”, where one pulse per second was skipped to increase the inter-pulse interval (IPI) and to study the ERNA in the time domain. After blocks of ‘skipped’ DBS, we applied ~ 20 bursts (consisting of 10 pulses at the frequency of the previous DBS block) spaced 1 s apart to study the post-DBS ERNA (Figure 2Ai). A third paradigm was tested in 9 participants (10 hemispheres, see Table 1), where we applied blocks of continuous (243.02 ± 29.29 s) and beta-triggered adaptive DBS (aDBS, 341.51 ± 53.99 s) as described before (Little et al., 2013). aDBS was applied such that the LFP was filtered around a pre-defined beta peak at rest ± 3 Hz, rectified and smoothed (over 400 ms) in real-time. Stimulation was triggered when the amplitude of beta band activity processed as described exceeded a certain threshold with a 250 ms ramp at the start and end of each burst (Figure 8A). The threshold was defined for each hemisphere at rest such that DBS was ON for ~ 50% of the time. Paradigm 1 and 2 were recorded ON medication (see Table 1); paradigm 3 was recorded OFF medication. Data recorded for previous studies with patients OFF levodopa (patients 15 to 23 and 25 to 26) are included to investigate the effect of medication (Wiest et al., 2021, 2020). If not indicated differently, a stimulation frequency of 130 Hz was used. Data from patients 1-17 (ON meds) and patients 15-26 (OFF meds) were included for the ON/OFF medication comparison in Figure 7.

### 2.5 Signal processing

All data analysis was performed using custom-written scripts in MATLAB (version 2020b, Mathworks, Massachusetts, USA). Continuous LFP signals were high-pass filtered at 1 Hz and notch-filtered at 50 Hz (second-order IIR notch filter). The ERNA was extracted from the time series waveform and the spectrogram. As any frequency analysis based on the time domain is strictly speaking an analysis in the frequency domain, we opted against the terms ‘time domain ERNA’ and frequency domain ERNA’ and chose to refer to our two approaches as ‘time series-based ERNA’ and ‘FFT-based ERNA’.

#### 2.5.1 Signal processing: Time series-based ERNA analysis

When stimulation is applied at 130 Hz, the IPI of ~ 7.7 ms only allows to study the latency and amplitude of the first ERNA peak. To do this, we removed a period of 1.2 ms after the positive deflection of the previous DBS pulse, as this was contaminated by artefact. Afterwards, we linearly detrended the signal during the IPI, applied a second-order low pass filter at 500 Hz and upsampled the IPI using a spline interpolation by factor 10. We defined the ERNA latency as the time between the previous DBS pulse (positive deflection of the artefact) and the first ERNA peak. The ERNA amplitude was defined as the absolute height of the first ERNA peak (as ERNA latencies increased towards the end of DBS blocks, the trough disappeared in some recordings, hence, we chose the absolute height of the peak to define the amplitude, Figure 2Av).

To test if the ERNA during stimulation was actually oscillatory, we skipped one DBS pulse per second during stimulation, which creates a larger window (~ 15.4 ms when DBS at 130 Hz) to study more than just the first ERNA peak (Figure 2Aii). Here, we processed LFPs during the IPI as described above and defined the ERNA frequency based on the distance between the first and second ERNA peak and the amplitude as the distance between the first ERNA peak and the next trough (Figure 2Aiv). The ERNA after bursts following the DBS blocks was analysed in a similar way (Figure 2Aiii).

#### 2.5.2 Signal processing: Fast Fourier transform (FFT)-based ERNA analysis

To compare this study with previously reported results, we also used an FFT-based approach to quantify the ERNA during continuous DBS. To this end, spectral amplitudes were estimated between 1 and 500 Hz using the short-time FFT with a window length of 1 s, 25% overlap of consecutive windows and a Hamming window as implemented in MATLAB’s *spectrogram* function yielding a frequency resolution of 1 Hz (Figure 2Bi). We calculated power spectral densities (PSD) of consecutive non-overlapping 5-s windows and tracked the largest peak between 200 and 450 Hz (as the ERNA peak) over time (Figure 2Bii). To avoid tracking harmonics of stimulation, we applied additional notch filters at 260 and 390 Hz. A similar method to quantify ERNAs in an FFT-dependent manner was used before (Wiest et al., 2020). ERNA frequency steady states were defined as in Wiest et al., 2020.

#### 2.5.3 Signal processing: Beta power analysis

Power change during DBS in Figure 5D was quantified relative to a 30 s baseline period before respective DBS blocks and the following formula:

$$\%power\;change=\frac{DBS\;power-baseline\;power}{baseline\;power}*100$$

for average beta (13-35 Hz) power. Thus, negative change corresponds to power suppression with stimulation and vice versa.

**Figure 3:**
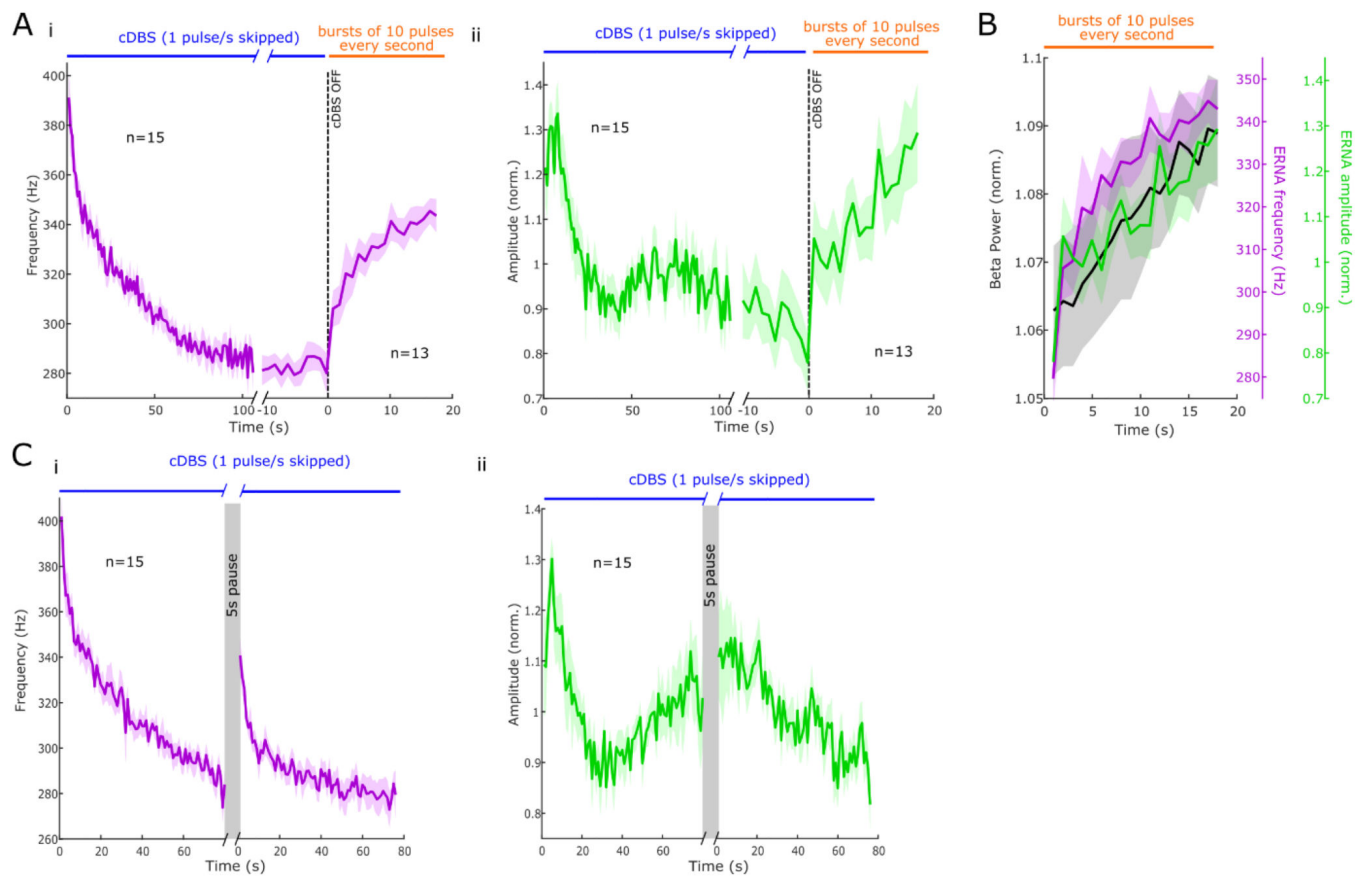
ERNA dynamics are relatively slow. **A.** Time series-based ERNA frequencies (i) and amplitudes (ii) during DBS initially decrease before reaching a steady state (mean ± SEM). When continuous DBS is turned off, ERNA steady state is not maintained, however, ERNA parameters need several seconds to return to baseline. **B.** The recovery of ERNA parameters and beta power after DBS happens at a similar rate. **C.** When pausing DBS for 5 s, ERNA frequencies (i) and amplitudes (ii) partially return to baseline values.

Beta power (13-35 Hz) recovery after DBS blocks (Figure 3B) was calculated based on the 1-s windows between consecutive bursts. For better temporal precision, we changed parameters of short-time FFT and used 500-ms windows with 50% overlap.

### 2.6 Statistics

Statistical analyses were conducted using custom-written scripts in MATLAB. To take repeated measures per hemisphere into account (hierarchical data), linear mixed-effect models were used to assess the effect of increasing DBS frequency and intensity on ERNA amplitudes and latencies. The ERNA features were set as dependent variable, the different frequency and intensity levels as fixed effects and the recorded hemispheres as repeated measures. The normal distribution of each variable and the residual were visually inspected with quantile-quantile plots and histograms of distribution. All models were estimated by the method of maximum likelihood and included random intercept for each hemisphere to capture individual differences. Changes of ERNA features between cDBS and aDBS were analysed in a similar way (Figure 8C). To identify clusters of significant differences between time series and FFT-based ERNA or ERNA ON and OFF medication, we used non-parametric permutation tests with 1000 permutations (Figure 2C and Figure 7A). To perform paired comparisons between different medication conditions, we used a paired samples permutation *t*-test with multiple comparison correction (50,000 permutations each; Figure 7C) as implemented in Groppe, 2022. When correlations were reported, we calculated Spearman’s rank coefficients because the non-baseline-transformed power data are non-normally distributed and contain outliers. Multiple statistical tests were performed in this study under FDR control at 5% using the adaptive linear step-up procedure, a modification of the original Benjamini and Hochberg procedure (see Duchet et al., 2021). This ensures that the expectation of the number of false positives over the total number of positives is less than 5% when many statistical tests are performed. All data are shown as mean ± standard error of the mean (SEM) unless mentioned otherwise.

## 3 Results

### 3.1 Time series and FFT-based ERNA dynamics follow similar slow dynamics during stimulation

A recent study suggested that high-frequency activity in the spectrogram during high-frequency DBS and evoked compound activity in the time domain were independent (Ozturk et al., 2021). Here, we directly compared the two activities in the same data set.

First, we defined ERNA frequencies based on the LFP waveform after every skipped pulse and found that their dynamics ON medication and their time to reach a steady state 75.25 ± 1.14 s are similar to the FFT-based ERNA frequency dynamics OFF medication described before (Figure 2C, Wiest et al., 2020). ERNA frequency dynamics quantified based on the times series and FFT (n = 15 hemispheres each) did not differ significantly (paired permutation *t*-test, p > .05, Figure 2C). To corroborate the link between the ERNA calculated based on time series and FFT, we found a strong positive correlation between time series and FFT-based ERNA frequencies (Spearman; *ρ* = .98, p < .001) and amplitudes (Spearman; *ρ* = .93, p < .001).

In addition to ERNA frequencies, we also quantified ERNA amplitudes over time (Figure 3Aii). ERNA amplitudes briefly increased after stimulation start before gradually decreasing to a steady state. ERNA amplitude dynamics, here shown for time series-based ERNA, are similar to what was previously shown in FFT-based ERNA (Wiest et al., 2020). In the rest of the manuscript (except Figure 7A) we focused on time series-based ERNA.

### 3.2 ERNA has slow dynamics after cessation of stimulation

To study if and how fast ERNA parameters return from steady state to baseline after DBS, which may reflect the recovery of the underlying network, repetitive DBS bursts (consisting of 10 pulses) were applied to probe the ERNA every second for ~ 20 s after continuous DBS was stopped. To reach steady state, continuous DBS was applied for 131.80 ± 5.80 s. As observed in Figure 3A, ERNA frequencies and amplitudes tended to progressively return to their starting values, following a similar time course (Spearman, *ρ* = .86, p < .001). The recovery of ERNA frequencies and amplitudes (and by inference the recovery of the underlying circuit) followed a similar time scale as beta power recovery (Figure 3B), as confirmed by significant positive correlations between beta power and ERNA frequencies (*ρ* = .96, p < .001) and ERNA amplitudes (*ρ* = .89, p < .001). Altogether, this suggests that these activities may reflect a related underlying process.

To confirm the slowness of ERNA dynamics, we paused continuous DBS for 5 s and hypothesised that this would be insufficient for complete recovery of ERNA parameters to baseline (Figure 3C). As predicted by Figure 3A, after pausing DBS for 5 s, both ERNA frequencies and amplitudes only partially returned to their starting values (86.75 ± 1.58% and 87.00 ± 5.53% of the starting values for frequencies and amplitudes, respectively).

### 3.3 ERNA has fast dynamics on a sub-second timescale

**Figure 4:**
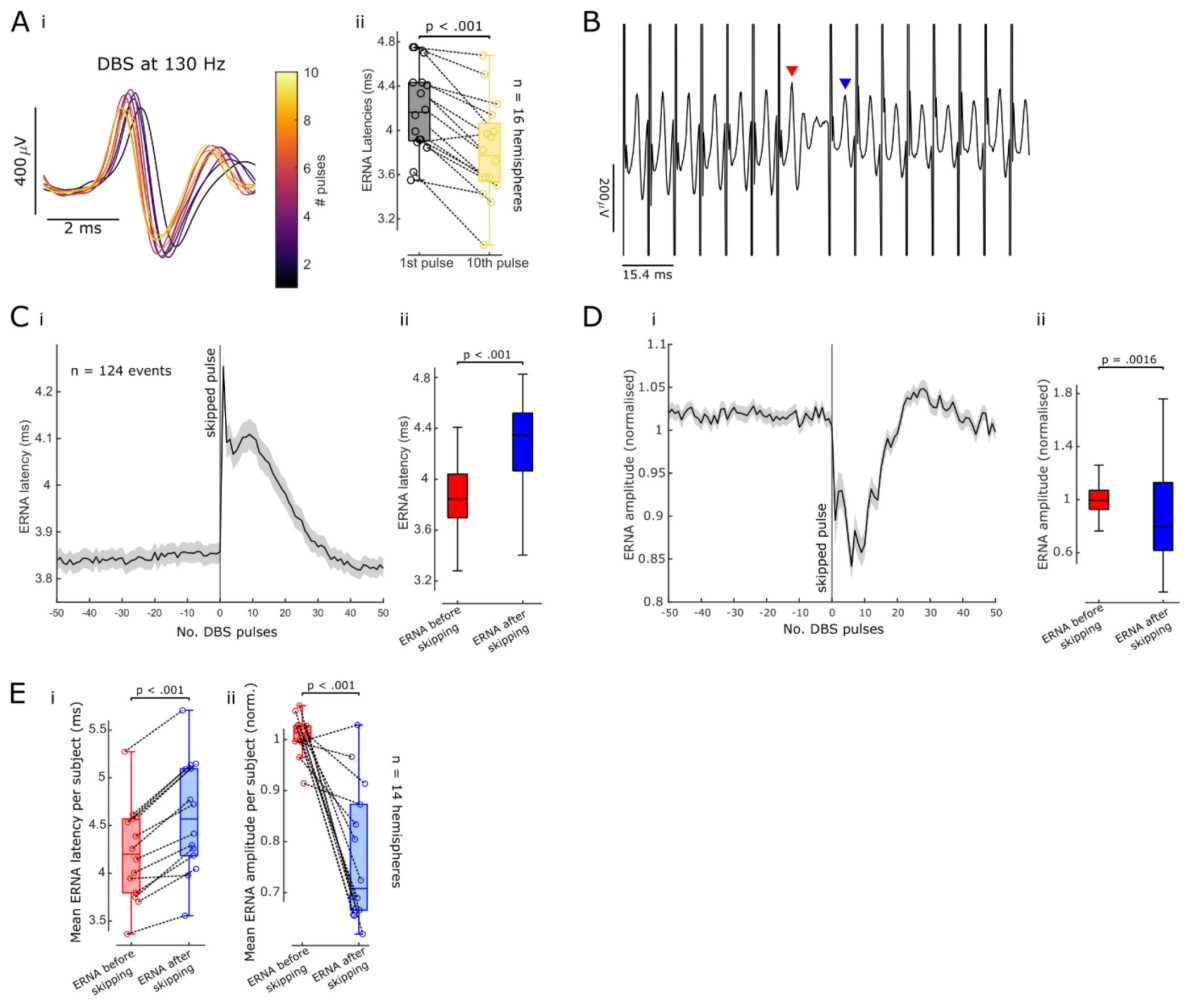
Fast ERNA dynamics. **A.** ERNA latencies and amplitudes change over the first 10 pulses (i, data from one example hemisphere shown). ERNA latencies decrease between the first and 10^th^ pulse (ii, paired-sample permutation *t*-test, p < .001). **B.** Skipping a single pulse affects ERNA parameters after the subsequent pulses (example time series shown, red and blue arrowheads denote the ERNA events that were analysed in C-E). **C.** ERNA latencies were aligned to the skipped pulse (mean ± SEM) over 124 events in one example hemisphere (i). ERNA latencies increase after skipping a single pulse in this subject (ii). **D.** ERNA amplitudes were aligned to the skipped pulse as in C (i). ERNA amplitudes decrease after skipping a DBS pulse (ii). **E.** When skipping one DBS pulse, average ERNA latencies increase (i, paired-sample permutation *t*-test, p < .001, n = 14 hemispheres), while average ERNA amplitudes decrease (ii, p < .001).

Analysing the ERNA in the time domain and quantifying its parameters after every DBS pulse allowed us to investigate its dynamics on a more refined time scale. In doing so, we found that in addition to the slow frequency and amplitude dynamics described above (Figure 3), the ERNA is modulated on a much faster scale. ERNA latencies and amplitudes change within the first 10 pulses (Figure 4Ai) and ERNA latencies decrease between the first and the 10^th^ pulse (paired permutation *t*-test, p < .001, Figure 4Aii).

The fast ERNA dynamics are also reflected in the changes after just a single pulse is skipped (Figure 4B). When aligned to the skipped pulse during DBS, ERNA latencies increased on average by 9.18 ± 0.92% compared to the previous DBS pulse (paired permutation *t*-test, t = -9.35, p < .001, Figure 4C+E) while ERNA amplitudes decreased by 23.32 ± 3.97% (t = 5.78, p < .001, Figure 4D+E). After about 30 pulses (~ 230 ms) this modulation wore off and ERNA frequencies and amplitudes returned to similar levels as observed before pulses were skipped (Figure 4C+D, for one example hemisphere). These fast dynamics, observed at the sub-second timescale, happened in addition to the slow ERNA dynamics over tens of seconds to a minute (Figure 3).

### 3.4 ERNA dynamics are modulated by different DBS frequencies

**Figure 5:**
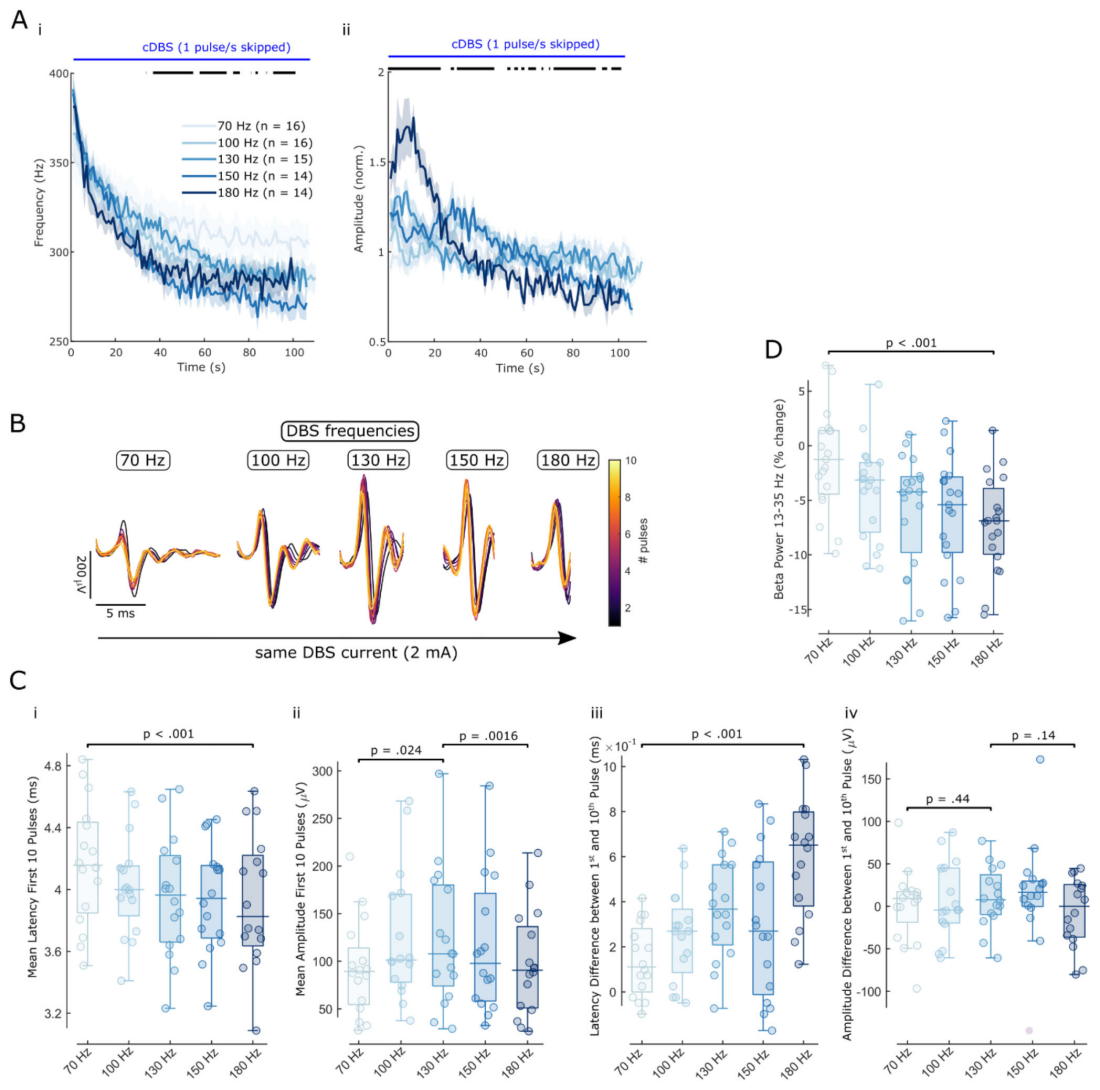
ERNA dynamics are modulated by different DBS frequencies. **A.** The slow dynamics of time series-based ERNA frequencies (i) and amplitudes (ii) are differently affected by increasing DBS frequencies over the first 100 s of stimulation. Bars above the plots denote significant clusters of a one-way ANOVA after multiple comparison correction. **B.** ERNA after the first 10 pulses of DBS blocks of increasing DBS frequency (shown for one example subject at 2 mA). **C.** Fast ERNA latency (i+iii) and amplitude (ii+iv) dynamics (of the first 10 pulses) are differently affected by increasing DBS frequencies. **D.** Beta power suppression during DBS relative to a 30 s baseline before the respective blocks becomes stronger with increasing DBS frequencies. C and D show p-values of linear mixed-effect models.

The above analyses focused on DBS at 130 Hz. To assess the impact of different DBS frequencies on slow and fast ERNA dynamics, we additionally applied stimulation at 70, 100, 150 and 180 Hz and analysed the ERNA in the time domain. First, we studied the slow ERNA dynamics over 2-min blocks of continuous DBS (Figure 5A). While ERNA frequencies were not significantly different at the start of the stimulation block across the 5 tested DBS frequencies, they diverged after ~ 20 s and stayed different until the end of the stimulation block. DBS at higher frequencies (150 and 180 Hz) led to lower ERNA frequency steady states compared to DBS at lower frequencies. ERNA amplitude dynamics at different DBS frequencies were more heterogeneous with most prominent modulation at 180 Hz.

Second, we investigated the effect of different DBS frequencies on the fast ERNA dynamics during the first 10 pulses (Figure 5B). The ERNA latency difference between the first and 10^th^ pulse increased from 70 to 180 Hz (Figure 5Ciii; LME: estimated effect of frequency = 9.69*10^-5^, t = 5.11, p < .001), whereas the ERNA amplitude difference between the same pulses did not differ across DBS frequencies (Figure 5Civ; LME: estimate = 4.75, t = 0.77, p = .44 from 70 to 130 Hz and estimate = - 8.65, t = -1.50, p = .14 from 130 to 180 Hz). The mean latency of the ERNA peak after the first 10 pulses gradually decreased with rising DBS frequency (Figure 5Ci; LME: estimate = -5.65*10^-5^, t = - 4.27, p < .001), and the mean amplitude of the first 10 ERNA peaks increased from 70 to 130 Hz (Figure 5Cii; LME: estimate = 15.21, t = 2.33, p = .024) and decreased from 130 to 180 Hz (LME: estimate = -13.19, t = -3.35, p = .0016).

To put the frequency-dependent effect of fast ERNA dynamics into context, we analysed the effect of increasing DBS frequencies on beta (13-35 Hz) power suppression (Figure 5D). To this end, we calculated beta power change during a given DBS block relative to a 30 s baseline period before respective DBS blocks and found that beta power suppression became more prominent as DBS frequencies were increased from 70 to 180 Hz (LME: estimate = -1.38, t = -5.24, p < .001). Beta power suppression with increasing stimulation frequency followed a similar pattern as average ERNA latencies of the first 10 pulses (Figure 5Ci), but there was no correlation between the two (Spearman, *ρ* = .06, p = .58).

### 3.5 ERNA dynamics are modulated with increasing DBS intensity

**Figure 6:**
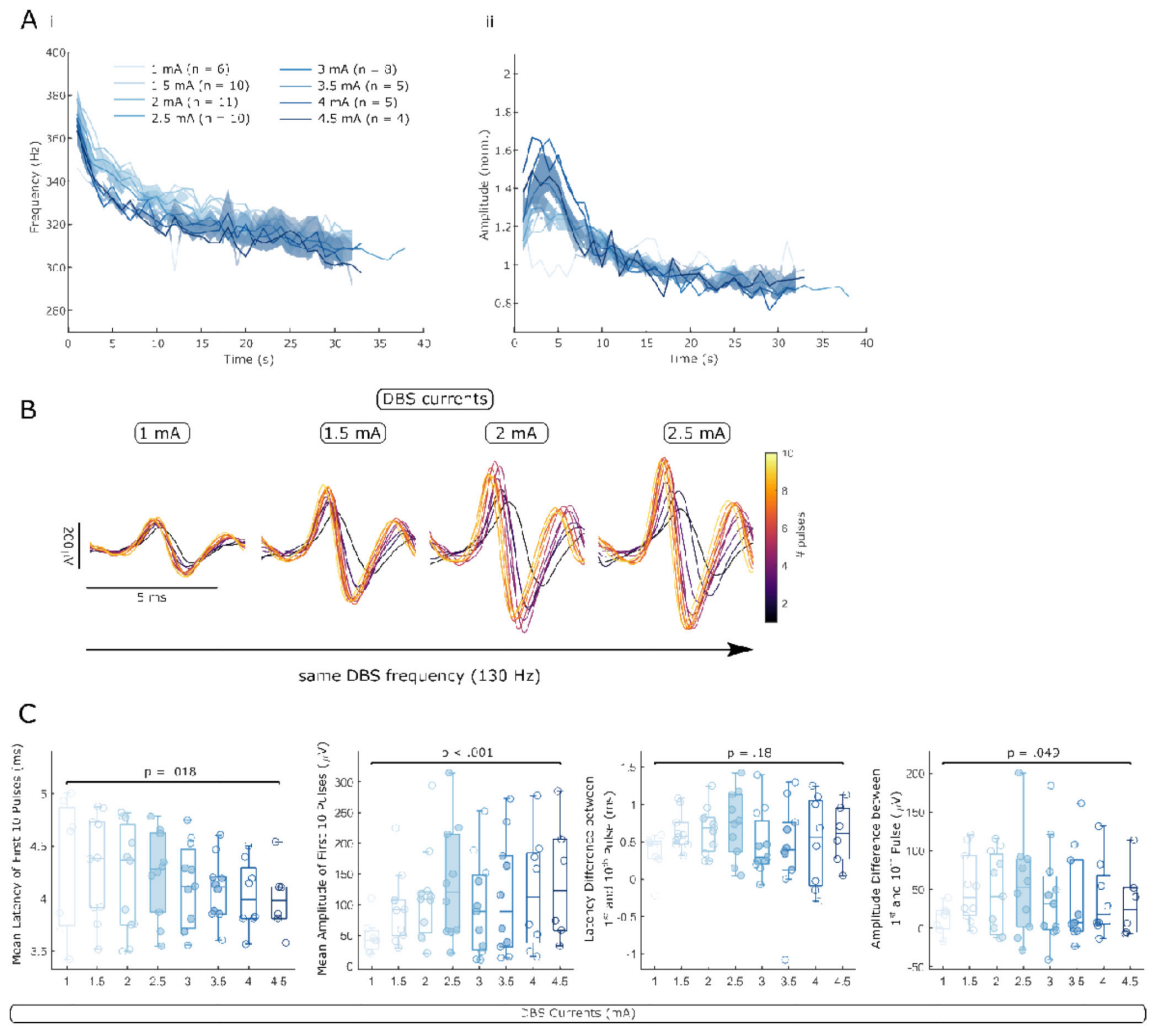
ERNA dynamics are modulated by different DBS intensity. **A.** The slow dynamics of time series-based ERNA frequencies (i) and amplitudes (ii) are differently affected by increasing DBS intensity over the first ~ 30 s of stimulation. After multiple comparison correction, no significant cluster of a one-way ANOVA survived. **B.** Fast ERNA dynamics are also modulated by increasing DBS intensity (ERNA after the first 10 pulses is shown for one example hemisphere). **C.** Mean ERNA latencies decrease and mean ERNA amplitudes increase with rising DBS intensity (in both cases the mean of the first 10 pulses). The ERNA amplitude difference between the first and 10^th^ pulse increases with rising DBS currents. C shows p-values of linear mixed-effect models.

Next, we studied the effect of different DBS intensities separately for slow and fast ERNA dynamics by applying 30 s blocks of cDBS at increasing intensity. While ERNA frequencies are not significantly different between the 8 intensity levels throughout the first 30 s, there is a trend that higher intensity elicits a steeper ERNA frequency drop resulting in lower values after ~ 5 s of DBS. ERNA amplitudes tend to differ between 2-7 s after DBS was switched on with larger amplitudes when higher intensity was used (Figure 6A). This tendency disappeared with prolonged stimulation (> 15 s).

Fast ERNA dynamics were equally affected by changing the DBS intensity (Figure 6B). Mean ERNA latencies of the first 10 ERNA events decreased (LME: estimate = -2.69*10^-5^, t = -2.42, p = .018) and mean ERNA amplitudes increased (LME: estimate = 23.12, t = 4.71, p < .001) when DBS intensity was increased from 1 to 4.5 mA (Figure 6C). The ERNA latency difference between the first and 10^th^ pulse was not affected by increasing DBS intensity (LME: estimate = 3.74*10^-5^, t = 1.37, p = .18), but the ERNA amplitude change across the first 10 pulses increased (Figure 6C; LME: estimate = 8.19, t = 2.00, p = .049).

### 3.6 ERNA dynamics are modulated by levodopa

**Figure 7:**
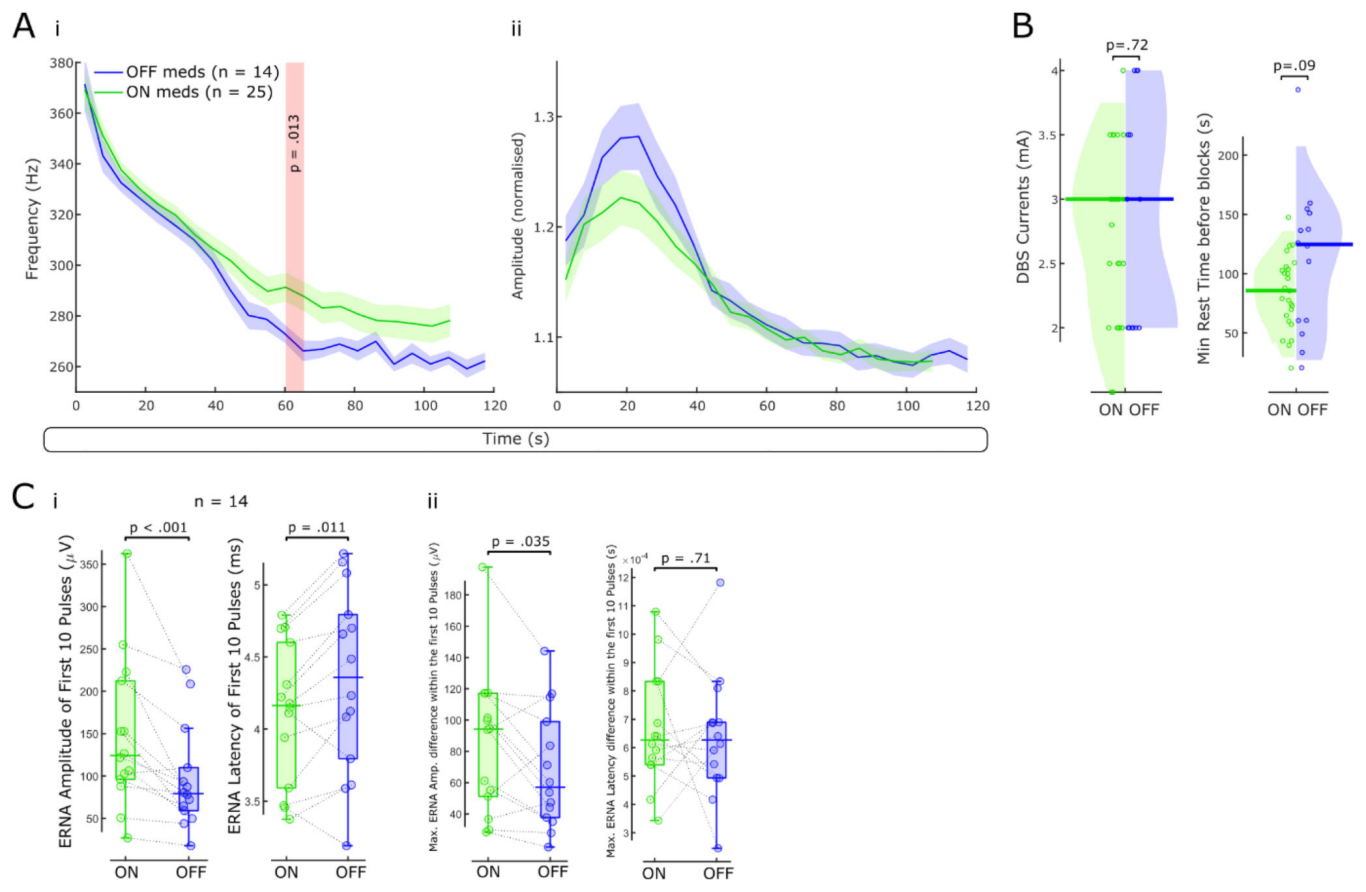
ERNA dynamics are modulated by levodopa. **A.** We first examined medication effects on the slow ERNA dynamics. FFT-based ERNA frequencies (i) and amplitudes (ii) are shown ON (n = 25 hemispheres) and OFF (n = 14) levodopa. **B.** DBS intensity and the rest time before DBS blocks did not differ between the ON and OFF levodopa groups. **C.** Mean ERNA amplitudes of the first 10 pulses of a DBS block (fast ERNA dynamics) are larger ON than OFF levodopa (paired samples permutation *t*-test, p < .001, n = 14 hemispheres). Mean ERNA latencies after the first 10 pulses are higher OFF medication (p < .011). The largest ERNA amplitude difference within the first 10 pulses is higher ON medication (p = .035); maximal ERNA latency differences across the first 10 pulses do not differ between both conditions.

Previously, ERNA parameters were linked with beta power and by inference parkinsonian symptoms (Schmidt et al., 2020; Sinclair et al., 2019; Wiest et al., 2020). Thus, we hypothesised that ERNA dynamics would differ with dopaminergic medication. We first studied the slow FFT-based ERNA dynamics ON and OFF levodopa during 130 Hz DBS. ERNA frequencies started at similar levels with and without levodopa, but ON medication they tended to reach steady state faster and at a higher frequency compared to the OFF medication state, yielding a significant difference after ~ 60 s (cluster-based permutation test, p = .013, Figure 7Ai). FFT-based ERNA amplitudes did not differ between both medication states (Figure 7Aii). DBS intensities and the resting time before respective DBS blocks did not differ between both groups (Figure 7B).

Furthermore, we tested if fast time series-based ERNA amplitude and latency dynamics over the first 10 ERNA events would differ between ON and OFF levodopa (Figure 7C). Mean ERNA amplitudes of the first 10 pulses were larger ON medication (paired samples permutation *t*-test, t = 3.87, p < .001, n = 14 hemispheres) and mean ERNA latencies were lower ON medication (paired samples permutation *t*-test, t = -3.14, p = .011). To highlight fast changes within the first 10 pulses, we calculated the maximum amplitude and latency difference. The maximal ERNA amplitude difference within the first 10 pulses was larger ON medication (t = 2.38, p = 0.035), while the largest latency difference did not differ between both conditions (t = 0.41, p = .71).

### 3.7 ERNA parameters differ between continuous and adaptive DBS

**Figure 8:**
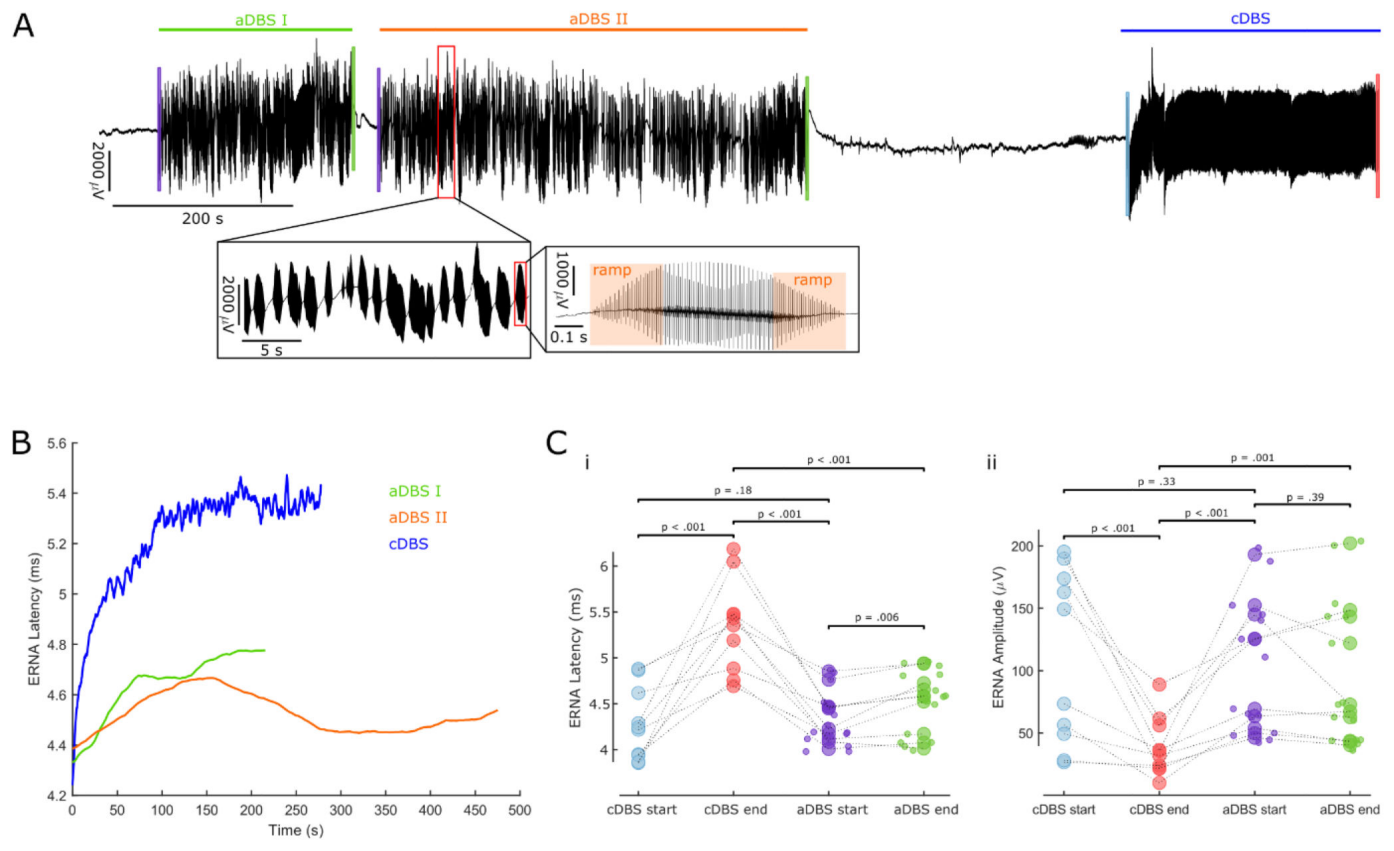
ERNA parameters differ between continuous and adaptive DBS. **A.** We recorded blocks of continuous DBS (130 Hz, blue) and beta burst-triggered adaptive DBS (DBS was switched on when beta activity exceeded a pre-defined threshold; green and orange) in the same subjects. The insets show a zoomed in view on DBS bursts. Note the 250 ms ramp on and off period. **B.** During cDBS, ERNA latencies and amplitudes were extracted after every DBS pulse (see Figure 2Av). During blocks of aDBS, only bursts > 0.5s were analysed and ERNA parameters were extracted after every pulse. We show ERNA latency dynamics during cDBS and aDBS (average values per burst) blocks in one representative hemisphere. **C.** ERNA latencies (i; p < .001) and amplitudes (ii; p < .001) during cDBS change from start (average of the first 10 pulses) to end (average of the last 10 pulses). Modulation with sustained aDBS is less pronounced (i; p = .006) or not present at all (ii; p = .39). (n = 10 hemispheres, small dots denote multiple aDBS blocks per hemisphere)

Continuous and beta burst-triggered adaptive DBS were shown to be at least equally effective in the past (Little and Brown, 2020). Here, we sought to compare ERNA dynamics between cDBS and aDBS. Within the same subjects, we stimulated continuously at 130 Hz and applied blocks of aDBS (Figure 8A). aDBS was applied when the band-pass filtered (around the beta peak at rest ± 3 Hz), rectified and smoothed (over 400 ms) LFP exceeded a threshold with a ramping period of 250 ms at the start and end of each burst. Thresholds were set such that DBS was ON ~ 50% of the time at rest. Subsequently, we extracted ERNA latencies and amplitudes after every DBS pulse of cDBS and aDBS (each burst separately, see Figure 2Av).

When mean ERNA latencies of all stimulation bursts in one aDBS block were plotted in sequence, we did not observe the same dynamics as during cDBS (Figure 8B, one representative hemisphere shown), due to the discontinuous, bursting nature of the stimulation. During aDBS, each stimulation burst lasted on average for 0.83 ± 0.007 s (bursts < 500 ms were excluded from analysis due to the ramping periods) and stimulation was switched ON for 48.86 ± 4.48% of the time. We then compared average ERNA amplitudes and latencies of the first and last 10 pulses of cDBS blocks with average ERNA parameters of the first and last burst of an aDBS block. While ERNA latencies increased during cDBS from start to end (LME: estimate = 1.08, t = 5.44, p < .001, n = 10 hemispheres), ERNA latencies during blocks of aDBS only showed a slight increase (LME: estimate = 0.16, t = 3.01, p = .006; Figure 8Ci). ERNA amplitudes of cDBS decreased from start to end (LME: estimate = -71.78, t = -4.02, p < .001), while ERNA amplitudes of aDBS were unaffected (LME: estimate = -6.81, t = - 0.88, p = .39; Figure 8Cii). The resting time before blocks of aDBS and cDBS (LME: estimate = 132.04, t = 1.49, p = .15) and duration of aDBS and cDBS blocks did not differ (LME: estimate = 98.49, t = 1.45, p = 0.16).

## 4 Discussion

In this study, we present three main findings. First, we show that the time series and FFT-based approaches are two separate ways of investigating the same activity, the ERNA. Second, we distinguish fast and slow ERNA dynamics and show how they are differently affected by DBS frequency, intensity and levodopa, which allows for speculations on the mechanisms underlying the ERNA. Third, we show that ERNA parameters differ between sustained aDBS and cDBS, which may point towards different states of the STN-GPe loop in both conditions.

### 4.1 Time series and FFT-based ERNA reflect the same activity

Previously, FFT-base ERNA (referred to as stimulation-induced high frequency oscillations (HFO)) and time series-based ERNA (referred to as evoked compound activity) were considered independent (Ozturk et al., 2021). This is contentious as by definition every activity in the time domain must have a representation in the frequency domain and vice versa. If the time series-based ERNA was not the time domain representation of FFT-based ERNA, we would expect to see another activity in the time frequency map that better mirrors the ERNA waveform. We show in this study that both time series and FFT-based ERNA frequencies have very similar time courses during the first 100 s of stimulation, which suggests they are caused by the same underlying process (Figure 2C). In addition, we found a strong positive correlation between time series and FFT-based ERNA amplitudes (see section 3.1), which was reported before (Ozturk et al., 2021). This tight correlation would be expected if FFT-based ERNA was identical with the time series-based ERNA.

Ozturk et al. attempted to separate evoked compound activity from HFOs by removing an average template in the time domain. They subsequently performed wavelet transformation and showed that residual signal still contained HFO (Ozturk et al., 2021). However, their residual still shows persistent stimulation artefact, which suggests the denoising is not completely effective. In this study, we show that ERNA dynamics change over time and contain complex dynamics, which explains why removing a constant template will not completely remove the ERNA. Furthermore, Ozturk et al. argue that 20 Hz DBS elicits ERNA in the time domain but not in the frequency domain and therefore both activities must be independent. We argue that 20 Hz stimulation does not elicit a clear ERNA in the frequency domain because the ERNA does not summate over consecutive DBS pulses, as it is too brief (ERNA at low frequency is damped earlier), inter-pulse intervals are too long and its amplitudes are lower. In addition, DBS at 130 Hz yields 130 separate ERNA events per second, which will result in higher amplitudes at ERNA frequency if a 1-s window is used for FFT. In comparison, 20 Hz DBS only yields 20 ERNA events per window and therefore a lower amplitude signal. Here, we show that stimulation at lower frequencies (e.g. 70 Hz) leads to a slower frequency decrease compared to higher DBS frequencies (Figure 5A). We hypothesise that the ERNA induced by stimulation at 20 Hz may not decrease in frequency at all over the first 22 s of stimulation and, hence, will not yield the stereotypical ERNA frequency drop in the spectrogram.

### 4.2 Mechanisms underlying the fast and slow ERNA dynamics

Previously, we reported the slow ERNA dynamics that reach a steady state after the first minute of cDBS (Wiest et al., 2020); dynamics that could be explained by synaptic failure (Denker and Rizzoli, 2010). Nevertheless, it was unclear whether ERNA steady state, and by inference the status of the neural network underlying the ERNA, was maintained after DBS is terminated and if so how long it would last. In this work, we corroborate the slow ERNA dynamics during DBS by analysing the ERNA in a time series-based approach and we demonstrate that ERNA frequency and amplitude steady states are not maintained once DBS is turned off. However, it takes several seconds for both parameters to reach starting values (Figure 3A). Such slow dynamics in the recovery period may be reconcilable with the time required to replenish presynaptic vesicle pools (Rizzoli and Betz, 2005). In addition, the partial recovery of ERNA parameters during a 5-s DBS pause speaks in favour of synaptic failure as underlying these slow dynamics (Figure 3C). The different slow ERNA frequency and amplitude dynamics at different DBS frequencies and intensities could also be explained by the synaptic failure hypothesis (Figure 5A+6A). The higher the stimulation frequency or current, the more pulses and energy are delivered, the steeper and faster is the ERNA frequency decrease. Accordingly, ERNA amplitude modulation was more pronounced at higher DBS frequencies and intensities.

Synaptic depletion could explain most of the above slow ERNA dynamics, but how exactly would that be the case? Recently, a computational model suggested the ERNA may be initiated by direct activation of cortico-subthalamic fibres and subsequently sustained by reciprocal STN-GPe connections (Schmidt et al., 2020). STN neurons and afferent and efferent axons in the STN may well be able to follow high-frequency stimulation for a prolonged period of time, however, synapses would most likely be depleted after a few seconds to minutes, resulting in functional disconnection of the STN from the basal ganglia network (Bergman, 2021). In that case, the ERNA could reflect STN intrinsic properties, however, the existence of functional intranuclear connectivity in STN is contentious (Steiner et al., 2019). We can further speculate on the origin of the ERNA. Direct activation of prototypic GPe terminals may lead to orthodromic activation of STN, which is likely obscured by the artefact in our recordings, and antidromic activation of GPe. GPe may then inhibit STN, yielding an ERNA deflection after ~ 4 ms, and inhibit itself. This auto-inhibition paired with its intrinsic pacemaker capabilities may produce the stereotypical ringing of the ERNA recorded in STN, which decreases in amplitude as GPe neurons become less synchronised. The ringing and time-locked firing pattern of pallidal neurons was described before (Bar-Gad et al., 2004). The ERNA is then propagated through the basal ganglia and can also be recorded from the internal globus pallidus (Awad et al., 2021).

In addition to the slow ERNA dynamics, we report fast dynamics at the start of stimulation (Figure 4A) and after pulses are skipped (Figure 4B-E). These fast ERNA dynamics are particularly interesting as they are modulated by levodopa (Figure 7C), and tend to be sensitive to changes in DBS frequency and intensity. We speculate that glutamate may have a modulatory effect on the ERNA. Fast glutamatergic hyperdirect pathway neurons were shown to be depressed after just a few pulses of high-frequency stimulation of the STN (Steiner et al., 2019). Medication-related differences over the first 10 pulses of stimulation could be explained by the levodopa effect on glutamatergic firing of these neurons. Given that fast ERNA dynamics are also sensitive to changes in DBS frequency and intensity suggests that these stimulation parameters may modulate glutamatergic, hyperdirect pathway inputs in STN. In contrast, slower GABAergic neurons were shown to be able to follow high-frequency DBS at moderate levels of depression (Steiner et al., 2019). The slow ERNA dynamics over the first minute of DBS may either be explained by synaptic failure of the glutamatergic STN-GPe synapse, which may have slower kinetics than the cortico-subthalamic synapses, or progressive synaptic depletion of GPe neurons.

Importantly, neither the synaptic depletion hypothesis, nor the origin of the ERNA between STN and GPe, nor the modulatory effect of glutamate have been proven beyond reasonable doubt and remain speculative.

### 4.3 The ERNA as a marker to predict the best DBS frequency and lowest effective intensity

Previous studies suggested ERNA amplitudes as a marker for DBS contact selection (Sinclair et al., 2018; Xu et al., 2022). Here, we observed that average ERNA amplitudes of the first 10 pulses change as a function of DBS frequency (Figure 5Cii), and are also modulated by dopaminergic medication (Figure 7C). In particular, average ERNA amplitudes of the first 10 pulses were larger ON medication compared to OFF medication and similarly, they were larger at 130 Hz, which most often yields the best clinical outcome. This suggests that ERNA amplitudes may be useful to predict the most effective frequency, however, the correlation with clinical benefits remains to be shown. These findings are consistent with previous studies showing that ERNA amplitudes increased from 5 to 130 Hz (Schmidt et al., 2020) and decreased from 130 to 180 Hz (Ozturk et al., 2021). This observation may be caused by resonance and positive interference between the ERNA and the next DBS pulse. It is possible that if the next DBS pulse is delivered during a sensitive phase of the ERNA oscillation, the next ERNA event is enhanced, while stimulation during a different phase (± 180°) might have the opposite effect (Ozturk et al., 2021). Inter-pulse intervals of 130 Hz DBS are ~ 7.7 ms long, which potentially corresponds to the sensitive period and may lead to augmented ERNA amplitudes. However, in clinical practice some patients report best symptom control at higher or lower DBS frequencies. It would be interesting to test in further studies if these patients have larger ERNA amplitudes at clinically more effective DBS frequencies.

Previously, beta and gamma power were shown to be suppressed by high-frequency DBS and were suggested as markers for placement and contact selection (Shah et al., 2022; Wiest et al., 2020). Here, we found increasing beta power suppression, a reduction of ERNA latencies and an increasing latency difference between the first and 10^th^ pulse while increasing DBS frequencies from 70 to 180 Hz (Figure 5). In contrast, ERNA amplitudes were not linearly modulated by rising DBS frequencies but revealed highest values at the frequency that empirically has best clinical effect. This may hint towards ERNA amplitudes as a better marker for DBS frequency selection than ERNA latencies and power suppression in the beta band.

Similar to DBS frequencies, ERNA parameters were modulated by changing DBS intensities. ERNA amplitudes increase with rising DBS intensity (Figure 6A+B), but in some hemispheres higher intensities were needed than in others to induce the same ERNA amplitudes. This likely relates to differences in placement (Wiest et al., 2022) and could be used to estimate the minimal effective DBS current. In future studies, this will have to be benchmarked against the DBS intensity used at the clinically most effective directional contact.

### 4.4 The ERNA is modulated in PD and may become a useful marker in aDBS

In the past, several attempts were undertaken to link ERNA features with parkinsonian symptoms. The time for ERNA amplitudes to reach steady state was linked with contralateral UPDRS scores (Wiest et al., 2020) and stimulation to the contact with highest ERNA amplitudes resulted in best UPDRS improvement (Sinclair et al., 2018). Given the strong link between beta activity and bradykinesia and rigidity (Kuhn et al., 2006), other groups linked ERNA features with beta power and by inference with parkinsonian symptoms. Beta power has been positively correlated with ERNA parameters after paired pulses (Awad et al., 2021), ERNA frequencies (Sinclair et al., 2019) and ERNA amplitudes over the first 10 s of DBS (Schmidt et al., 2020). Here, we expand on this and report levodopa-induced changes of fast ERNA dynamics (Figure 7C). Mean ERNA amplitudes of the first 10 pulses are higher ON medication, while mean ERNA latencies are lower ON medication, in keeping with increased frequencies. This change may be due to plastic changes in the dopamine-deficient brain (Alberquilla et al., 2020; Smith et al., 2009), which affects reciprocal STN-GPe connections. If the ERNA was a reflection of the STN intrinsic properties, ERNA changes with medication might reflect changes of the STN intrinsic properties due to STN dopaminergic receptors (Galvan et al., 2014). Alternatively, it is possible that the reduced overall functional cortico-striatal connectivity in the dopamine-deficient state leads to reduced resonance and lower ERNA amplitudes (Baggio et al., 2015; Vancea et al., 2019). In addition, we found a significant difference between ERNA frequencies ON and OFF medication after ~ 1 min, which might hint at different times to reach steady state and potentially a steady state at higher frequencies ON medication (Figure 7Ai). Furthermore, we found a link between ERNA parameters and beta power after DBS is switched off (Figure 3B). This may indicate that ERNA and beta power in the dopamine-deficient brain are modulated by the same underlying process.

These results together suggest that the fast ERNA dynamics, mostly ERNA amplitudes, could act as a biomarker for parkinsonian symptoms and may be useful in aDBS paradigms. In this context, it may be possible to calculate ERNA parameters of aDBS bursts in real-time and extract information about medication states or fast motor fluctuations and in turn adapt the threshold to trigger aDBS.

### 4.5 cDBS and aDBS may lead to differences in the synaptic status of the cortico-subthalamic-GPe circuit

aDBS was shown to be more effective than sham stimulation or no stimulation and at least as effective as cDBS with fewer side effects (Little et al., 2013; Little and Brown, 2020). While the exact mechanism underlying aDBS is unclear, it is believed to specifically target long beta bursts, which are linked with bradykinetic movements (Tinkhauser et al., 2017; Torrecillos et al., 2018). Here, we show that ERNA parameters are differently modulated during cDBS and aDBS (Figure 8C), while cDBS and aDBS equally improved motor performance compared with no DBS (He et al., 2022). While both ERNA amplitudes and latencies drift towards a steady state in cDBS, we did not observe that drift in aDBS due to the discontinuous burst stimulation during aDBS. If the slow ERNA dynamics during cDBS are caused by synaptic failure, it is conceivable that synapses have ample time to replenish between aDBS bursts and therefore never deplete completely and never reach a steady state. While aDBS was at least as effective as cDBS in this cohort and previous studies (He et al., 2022; Little and Brown, 2020), we hypothesise that different network states could cause clinical improvement in both instances. More studies will be needed to explore if the difference in the synaptic status during aDBS compared with cDBS, as revealed by the ERNA, could explain why aDBS can help reduce side effects and preserve physiological function of the cortico-subthalamic-GPe circuit. However, this is still speculative. cDBS has been extensively proven as an effective long-lasting treatment of advanced PD and if aDBS, which may elicit a different synaptic state of the STN-GPe circuit than cDBS, will have the same long-term clinical effects, still remains to be tested.

### 4.6 Limitations

The major limitation of this work is the lack of correlation between ERNA parameters and clinical outcome. We speculated that ERNA amplitudes may aid the selection of DBS frequency and intensity. We will test this in a larger follow-up cohort, where we would like to correlate the DBS frequency that elicits largest ERNA amplitudes with the frequency of best clinical efficacy. Similarly, we would like to test if the lowest DBS intensity that elicits ERNA will also be the lowest effective clinical intensity and finally, if an ERNA-based adaptive feedback algorithm will be complementary to a beta-only aDBS algorithm.

Another limitation of this study is that we cannot infer the origin of the ERNA at the cellular level from our data. We speculated on the potential origin of the ERNA, but we acknowledge that these considerations are only supported by computational studies thus far and, hence, remain speculative. Further studies with simultaneous LFP and multi-unit activity recordings across the basal ganglia (STN and GPe) would be required to test our hypotheses.

## 5 Conclusion

This study provides evidence that time series and FFT-based ERNA describe the same activity. We further describe the fast and slow ERNA dynamics during and after DBS blocks, how these are modulated by different DBS frequencies, intensities and medication states and speculate on how these dynamics may be caused by synaptic failure. We report different ERNA dynamics during cDBS and aDBS, which together with the marked difference of the fast ERNA dynamics ON levodopa, might create a basis for the ERNA as a complimentary feedback marker in aDBS that signals changes in medication or motor fluctuations in real-time. We speculate on the origin of the ERNA and our results are in keeping with the ERNA in STN LFPs reflecting rhythmic inhibitory input from GPe.

## Figures and Tables

**Table 1:** Clinical and recording details. UPDRS: Unified Parkinson’s Disease Rating Scale, FOG: freezing of gait, dir: directional, L: left, R: right, aDBS: adaptive deep brain stimulation, SG: St. George’s Hospital, K: King’s College Hospital, M: University Medical Centre Mainz, STN: subthalamic nucleus.

Patient #	Gender (m/f)	Age (yr)	Disease Duration (yr)	UPDRS-III OFF meds (pre-OP)	UPDRS-III ON meds (pre-OP)	Predominant Symptoms	Time of Recording (days post-OP)	DBS system	STN tested (L/R)	DBS Intensity (mA)	Meds state	Skipped pulse paradigm	aDBS (paradigm 3) (L/R)	Site
1	m	54	6	51	35	tremor, bradykinesia	5	Boston Cartesia HX	L+R	L: 4 R: 3	ON	yes	no	SG
2	m	58	11	41	16	tremor, bradykinesia	5	Boston dir	L+R	L: 2.5 R: 2	ON	yes	no	SG
3	m	60	6	31	4	motor fluctuations, dyskinesia	5	Boston Cartesia X	L+R	L: 3 R: 2	ON	yes	R	SG
4	f	64	10	29	6	bradykinesia, motor fluctuations	5	Medtronic SenSight	L+R	L: 1.5 R: 1.5	ON	yes	R	SG
5	m	63	20	51	27	tremor, motor fluctuations	4	Boston Cartesia X	L+R	L: 2.5 R: 2.5	ON	yes	L+R	SG
6	f	64	7	29	8	bradykinesia, motor fluctuations	4	Medtronic SenSight	L+R	L: 2.5 R: 2	ON	yes	no	SG
7	f	67	6	60	17	tremor, bradykinesia	4	Medtronic SenSight	R	3	ON	yes	no	K
8	m	71	8	67	12.5	tremor, FOG	3	Medtronic model 3389	L	3.5	ON	yes	no	K
9	f	64	15	26	13	gait disturbance, bradykinesia, motor fluctuations	5	Medtronic SenSight	L	2	ON	yes	L	SG
10	f	66	6	16	6	tremor, bradykinesia, rigidity	5	Medtronic SenSight	R	2	ON	yes	R	SG
11	m	60	15	47	13	tremor	4	Medtronic SenSight	L	3.5	ON	yes	L	K
12	m	63	13	46	13	motor fluctuations, dyskinesia, unpredictable OFF periods	6	Medtronic model 3389	L	3	ON	no	L	K
13	f		22	48	5.5	dyskinesia, motor fluctuations	4	Abbott dir	L	3.5	ON	no	no	K
14	m	67	6	61	26	tremor	6	Medtronic SenSight	L+R	L: 3.5 R: 3.5	ON	no	L	K
15	m	65	5	34	16	FOG, motor fluctuations	4 and 5	Boston dir	L+R	L: 3 R: 2.5	ON+OFF	no	no	SG
16	m	64	13	52	21	FOG, motor fluctuations	4	Boston dir	R	2	ON+OFF	no	no	SG
17	m	53	7	23	12	tremor, bradykinesia	4 and 5	Boston dir	L+R	L: 3 R: 2	ON+OFF	no	no	SG
18	m	51	5	27	13	bradykinesia, rigidity, motor fluctuations	4	Boston dir	R	4	OFF	no	no	SG
19	m	60	15	50	30	FOG, motor fluctuations	3	Boston dir	L	2	OFF	no	no	SG
20	f	63	11	40	17	FOG, motor fluctuations	4	Boston dir	L	2	OFF	no	no	SG
21	m	47	16	71	37	rigidity,FOG, tremor	4	Boston dir	R	2	OFF	no	no	SG
22	m	53	7	38	25	tremor	5	Boston dir	R	3.5	OFF	no	no	SG
23	m	55	16	51	19	rigidity, bradykinesia	4	Medtronic model 3389	L	4	OFF	no	no	SG
24	m	69	20	37	18.5	motor fluctuations, camptocormia	n.a.	Medtronic model 3389	L	n.a.	OFF	no	no	K
25	m	61	10	24	12	akinetic-rigic with tremor	3	Medtronic model 3389	L	4	OFF	no	no	M
26	m	61	11	16	7	akinetic-rigid with motor fluctuations	2	Abbott dir	L	3.5	OFF	no	no	M
27	f	48	6	73.5	30	rigidity, dyskinesia	3	Abbott dir	R	n.a.	OFF	no	R	K

## Data Availability

All data will be made available via the MRC BNDU Data Sharing Platform (https://data.mrc.ox.ac.uk/) from the corresponding author upon reasonable request.
